# Description of a New Marine Cyanobacterium from the Cabo Verde Archipelago: Pigments Profile and Biotechnological Potential of *Salileptolyngbya caboverdiana* sp. nov.

**DOI:** 10.3390/md24010029

**Published:** 2026-01-08

**Authors:** Aimone Jussiene Cardoso Duarte, Guilherme Scotta Hentschke, Flávio Oliveira, Vitor Vasconcelos, Graciliana Lopes

**Affiliations:** 1 CIIMAR/CIMAR LA, Interdisciplinary Centre of Marine and Environmental Research, University of Porto, Terminal de Cruzeiros do Porto de Leixões, Av. General Norton de Matos s/n, 4450-208 Matosinhos, Portugal; aimoneduarte@gmail.com (A.J.C.D.); oliveira_flavio@outlook.pt (F.O.); glopes@ciimar.up.pt (G.L.); 2 Department of Biology, Faculty of Sciences, University of Porto, Rua do Campo Alegre 1021, 4169-007 Porto, Portugal

**Keywords:** carotenoids, taxonomy, new species, phycobiliproteins, 16S rRNA gene, toxins

## Abstract

Cyanobacteria are prolific producers of specialized metabolites of growing interest for blue biotechnology, transversal to various sectors such as cosmetics, foods and pharmaceuticals. In this work, the marine cyanobacterial strain *Salileptolyngbya* sp. LEGE 181209, from Cabo Verde, was systematically characterized to resolve its taxonomy, pigments profile, and cytotoxicity assessment. A polyphasic workflow combining 16S rRNA gene phylogenies, 16S–23S ITS secondary structures, *p*-distance, morphology, and scanning electron microscopy (SEM) was used to establish the taxonomic placement of the strain as a new species of the genus. PCR assays targeting the toxin biosynthetic genes *mcyA* and *anaC*, and cytotoxicity assays in HaCaT keratinocytes showed low-to-absent cytotoxicity, supporting a safety-forward profile for downstream use. A sequential extraction with solvents of different polarities yielded complementary pigment fractions profiled by HPLC-PDA and spectrophotometry. Total carotenoids reached 72.7 µg mg^−1^ of dry extract (DE), the profile being dominated by β-carotene and zeaxanthin (≈42 and 8 µg mg^−1^ of DE, respectively); chlorophyll-*a* was also very representative, reaching 85.6 µg mg^−1^ of DE. Phycobiliproteins dominated the polar fraction, with phycocyanin reaching 150 µg mg^−1^, followed by sugars (19.7 µg of glucose equivalents mg^−1^) and phenols (8.8 µg of gallic acid equivalents mg^−1^).

## 1. Introduction

Cyanobacteria and microalgae are ancient architects of life on Earth, having played a pivotal role in shaping the planet’s atmosphere and sustaining early ecosystems [1]. These photosynthetic prokaryotes, belonging to the domain *Bacteria*, are Gram-negative, and the only known prokaryotes capable of performing oxygenic photosynthesis, a turning point in Earth’s history [2]. Their ecological success derives from structural and physiological plasticity, including the presence of chlorophyll-*a* and accessory pigments organized into thylakoid membranes and phycobilisomes [3,4]. Many species also exhibit unique adaptations such as filament branching, hormogonia formation, and asymmetrical cell division, contributing to their resilience across diverse environments [5,6].

Cyanobacteria inhabit a wide variety of habitats, from freshwater and marine ecosystems to soils, and extreme environments, such as hot springs and polar regions [7,8]. However, their diversity remains underexplored, particularly in remote oceanic islands such as Cabo Verde [9]. Located in the central Atlantic Ocean, Cabo Verde comprises ten volcanic islands with an Exclusive Economic Zone of nearly 800,000 km^2^ [10]. The combination of tropical conditions, semi-arid climate, and nutrient-limited waters creates diverse ecological niches, supporting distinctive microbial communities [11]. The exploration of this biodiversity aligns with the country’s growing interest in blue economy and the valorization of marine resources. Taxonomic research is thus essential to understand cyanobacterial diversity and to enable the discovery of new bioactive producers [12,13]. Traditional morphology-based classification has given way to polyphasic approaches integrating molecular, morphological, and ecological data [14]. Among these, 16S rRNA and ITS sequencing provide robust phylogenetic resolution, allowing the distinction of closely related taxa and the description of new species [12]. Accurate identification underpins biodiversity assessment and the search for promising biotechnological candidates [15].

Cyanobacteria’s ecological versatility is largely linked to their ability to produce a wide array of specialized metabolites that function as adaptive responses to environmental stressors such as ultraviolet radiation, salinity, and oxidative stress [16]. These metabolites include peptides, alkaloids, polyketides, phenolics, terpenes, and polyunsaturated fatty acids [17], many of which display antimicrobial, antiviral, anti-inflammatory, antioxidant, or cytotoxic properties [18,19]. Consequently, cyanobacteria are increasingly regarded as sustainable sources of natural bioactive molecules with applications in medicine, cosmetics and food industries [20]. Among these metabolites, pigments play multifunctional roles, sustaining photosynthesis and photoprotection, and also contributing to antioxidant and anti-aging mechanisms [21]. Carotenoids and phycobiliproteins (PBPs) are the most prominent pigment groups. Carotenoids, hydrophobic tetraterpenoids divided into carotenes and xanthophylls, protect cells against photooxidative stress and display antioxidant, photoprotective, and anti-inflammatory properties [22,23]. PBPs, on the other hand, are water-soluble chromoproteins that extend photosynthetic efficiency and provide vivid coloration due to their phycobilin chromophores [16,24,25]. The main PBPs, phycocyanin (PC), phycoerythrin (PE) and allophycocyanin (APC), are highly valued for their fluorescence and antioxidant potential, supporting applications as natural colorants and bioactive ingredients. Marine cyanobacteria, particularly from tropical regions, are rich in these pigments, positioning them as key resources for blue biotechnology [9,11,26]. Despite this potential, marine strains remain poorly explored, especially in Cabo Verde. Within this framework, members of the genus *Salileptolyngbya* have drawn particular attention. These filamentous cyanobacteria typically inhabit saline or coastal environments. Some strains have been associated with pigment fingerprints dominated by β-carotene and zeaxanthin, as well as high PC yields, reflecting adaptations to strong irradiance and oxidative stress [19,20,26]. However, few *Salileptolyngbya* species have been described to date, and their biochemical traits remain poorly characterized.

In this study, we describe a new marine cyanobacterium isolated from the coastal environment of Cabo Verde, *Salileptolyngbya caboverdiana* sp. nov. LEGE 181209. A polyphasic approach combining morphological observations, scanning electron microscopy (SEM) and 16S rRNA/ITS phylogenetic analyses were applied to determine its taxonomic position. Solvents of different polarities enabled the characterization of its pigment profile by HPLC-PDA and spectrophotometry. Cytotoxicity assessment in HaCaT keratinocytes was also performed to assess safety and potential downstream application.

## 2. Results and Discussion

### 2.1. Taxonomic Results

**Figure 1:**
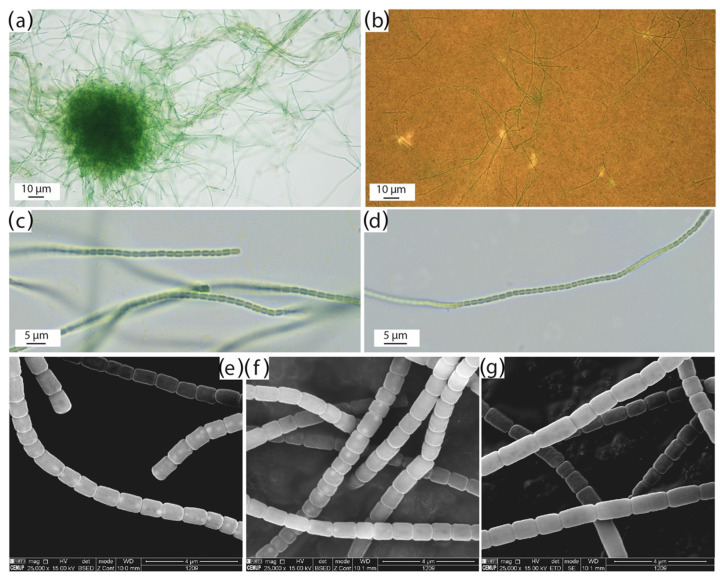
Light and scanning electron micrographs of *S. caboverdiana* sp. nov. LEGE 181209. (**a**) Light microscopy showing a dense aggregate of entangled filaments in liquid culture (10 µm). (**b**) Slide preparation with China ink, showing the absence of diffluent mucilage surrounding the trichomes (10 µm). (**c**,**d**) Light-microscopy details of non-attenuated trichomes displaying clear cross-wall constrictions and homogeneous cell content (10 µm). (**e**,**f**) Scanning electron micrographs with BSED filter showing trichomes with bright spherical inclusions, corresponding to internal pseudovacuoles (4 µm). (**g**) Scanning electron micrograph of trichomes with ETD filter (4 µm).

The strain LEGE 181209 was studied using polyphasic approach [6]. Under light microscopy, this strain is morphologically very similar to the existent species of *Salileptolyngbya*, namely *S. diazotrophica* Zhou & Ling [27] and *S. insularis* Araújo et al. [28], exhibiting trichomes with non-attenuated ends, distinct constrictions at the cross-walls, and comparable cell dimensions. However, LEGE 181209 can present isodiametric cells, while the other species of the genus presents cells that are always longer than their width. It also differs from *S. diazotrophica* by the absence of firm sheaths, and differs from *S. insularis* by the absence of translucent septa between cells. Additionally, SEM revealed conspicuous internal pseudovacuoles, with a high content of Carbon and Phosphorus (Figure 1 and Figure S1), located primarily next to the cells cross-walls, and which were never observed in *S. diazotrophica* and *S. insularis* (Figure 1, Table 1). This type of pseudovacuole has previously been reported in *Vacuolonema* Hentschke & Morais [29], and although these structures may resemble granules in SEM analysis, this strain is morphologically very similar to the existent species of *Salileptolyngbya*, namely *S. diazotrophica* Zhou & Ling. Analysis of *Vacuolonema* revealed them to be low-density regions, indicating the presence of empty spaces, forming pseudovacuoles. To date, these structures appear to be unique to *S. caboverdiana* sp. nov., as SEM and TEM analyses of *S. diazotrophica* showed no evidence of their presence [27]. In *S. insularis*, TEM images revealed the presence of pseudovacuoles [28]; however, only a single pseudovacuole per cell was observed, and it was not located adjacent to the cell cross-walls as in *S. caboverdiana*. Together with *Vacuolonema*, this represents the second report of this pseudovacuole arrangement. Given that these structures are detectable only by electron microscopy, we suspect that similar features may occur in other cyanobacterial genera but have not yet been observed or reported. Ecologically, LEGE 181209 is also distinct, being the only epilithic species of the genus, while the others are planktonic (*S. diazotrophica*) or benthic–epipsamic (*S. insularis*).

**Figure 2:**
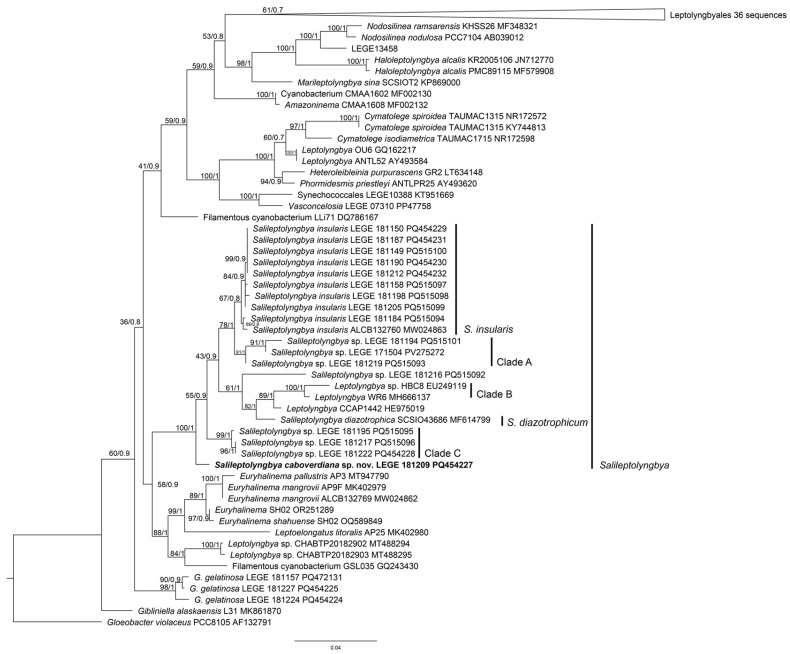
Maximum Likelihood phylogenetic tree of 16S rRNA genes, including LEGE 181209 (bold) and related species. ML and Bayesian Inference (BI) support values are shown at the nodes.

Phylogenetically, the Maximum Likelihood (ML) and Bayesian Inference (BI) trees (Figure 2) place LEGE 181209 within a strongly supported *Salileptolyngbya* clade (ML = 100, BI = 1), confirming its classification in this genus of the order Nodosilineales. However, this strain is phylogenetically distant from the type species *S. diazotrophica* and *S. insularis*, forming a separate branch at the base of the genus clade and indicating that it is a distinct species needing formal description.

The 16S rRNA gene identity matrix (Table S1) also confirms that LEGE 181209 is distinct from the other described *Salileptolyngbya* species, sharing a maximum of only 98% sequence identity with *S. insularis* ALCB132760. Identity values below 98.7% are strongly indicative of species separation [30]. This molecular divergence is further supported by the high 16S–23S ITS dissimilarity with the type species *S. diazotrophica*, which reaches 9.8%, also strongly indicating species separation [31].

**Figure 3:**
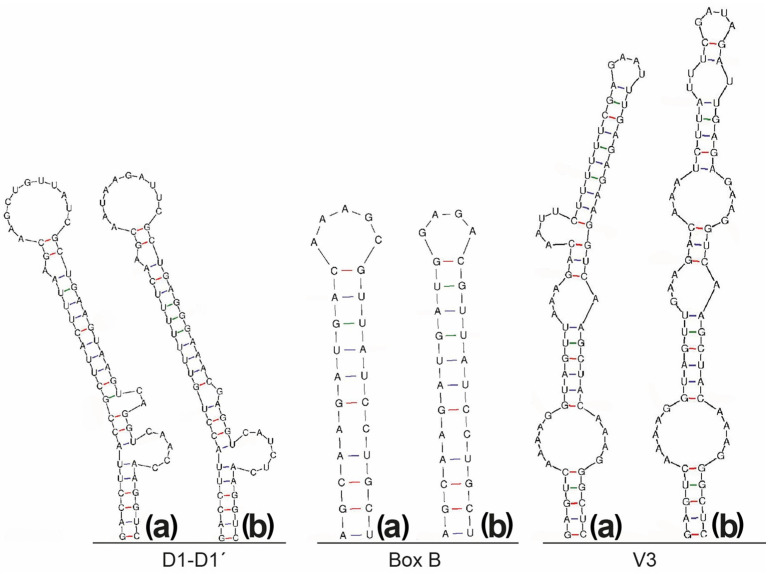
Predicted secondary structures of the 16S–23S ITS regions (D1–D1′, Box B and V3 domains) of (**a**) *Salileptolyngbya* sp. LEGE 181209 compared with (**b**) *S. diazotrophica*, showing clear structural differences supporting species-level separation.

The 16S–23S ITS secondary structures further support the divergence of LEGE 181209 from other *Salileptolyngbya* species (Figure 3). The D1–D1′ helix of LEGE 181209 differs from *S. diazotrophica* in sequence, length, and overall structure. For example, the unilateral basal bulge of LEGE 181209 contains the sequence 5′CAACC3′, whereas *S. diazotrophica* presents 5′CAUCUC3′. The middle stems of the D1–D1′ helix are different between the two species, with LEGE 181209 presenting two mismatches in the 3′ side of the molecule (“CA”), whereas *S. diazotrophica* presents only a guanine. The Box B helix of LEGE 181209 exhibits a larger terminal and different sequence from *S. diazotrophica*. The V3 helix is the most conserved among the structures; however, notable differences are observed in the terminal regions of these helices. The V2 region is absent in both strains’ sequences. Regarding the comparisons of the 16S–23S ITS region, no sequence of this region is available for *S. insularis* in public databases. Although these structures are presented in Araújo et al., [28], they seem to be not correctly constructed, considering that the D1–D1′ helix does not present the typical structure with a unilateral basal bulge.

Considering the results obtained through a polyphasic approach, which included standard parameters for species identification in cyanobacteria [6]—such as 16S rRNA gene ML and BI phylogenies, molecular identity (*p*-distance), 16S–23S ITS secondary structures, and morphological comparisons—it is evident that LEGE 181209 represents a distinct species of *Salileptolyngbya*, as described below.

#### *Salileptolyngbya caboverdiana G. S. Hentschke* and *F. Oliveira* sp. nov.

Diagnosis: Differs from the other species of the genus by presenting isodiametric cells and pseudovacuoles. Differs from *S. diazotrophica* by the absence of firm sheaths. Differs from *S. insularis* by the absence of translucent septa between cells.

Description: In culture, filaments are straight or curved, and solitary or entangled. Trichomes are constricted at the cross-walls. Cells are isodiametric, longer or shorter than their width, 1.3–1.9 μm long × 1.35–1.60 μm wide. Cell content is homogeneous under light microscopy. Under electron microscopy, pseudovacuoles are conspicuous next to the cells cross-walls.

Etymology: The species epithet “*caboverdiana*” refers to Cabo Verde, the country where the type strain was collected.

Holotype: Collected from a biofilm on rocks of a pier wall at Ponta do Sol, Santo Antão, Cabo Verde (17° 12.28′ N, 25° 05.53′ W), by Vitor Ramos, Jorge Neves, Pedro Leão, João Morais and Vitor Vasconcelos, 2018. Deposited in the University of Porto herbarium, in metabolically inactive state (lyophilized), under the code PO-T5291.

Reference strain: LEGE 181209 (16S rRNA gene, GeneBankPQ454227).

Habitat: Marine, growing on rocks of a pier wall.

### 2.2. Chemical Profile

#### 2.2.1. Carotenoid and Chlorophylls Profiling by HPLC-PDA

**Figure 4:**
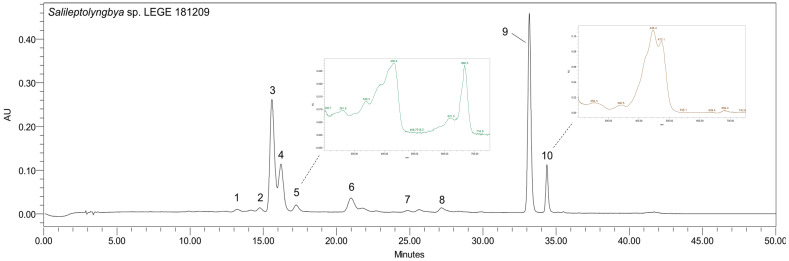
Carotenoid and chlorophylls profile of the acetonic extract of *S. caboverdiana* sp. nov. LEGE 181209. HPLC-PDA recorded at 450 nm, highlighting the UV–VIS spectra of compounds 5 and 10. Identified pigments: lutein (1), oxygenated derivative of β-carotene (2), chlorophyll-*a* (3), zeaxanthin (4), chlorophyll-*a* derivative (5), myxoxanthophyll (6), β-cryptoxanthin (7), β-carotene derivative (8), β-carotene (9), and β-carotene derivative (10).

The analysis of the acetonic extract of *S. caboverdiana* sp. nov. LEGE 181209 by HPLC-PDA enabled the identification of ten pigments: eight carotenoids, chlorophyll-*a* and a chlorophyll-*a* derivative. The identified pigments included four xanthophylls (lutein (1), zeaxanthin (4), myxoxanthophyll (6), and β-cryptoxanthin (7)), one carotene (β-carotene (9)) and chlorophyll-*a* (3). In addition, four unidentified carotenoids were detected: an oxygenated derivative of β-carotene (2), two β-carotene derivatives (8, 10) and a chlorophyll-*a* derivative (5), which displayed spectral characteristics similar to authentic standards but distinct retention times. The adopted methodology allowed a good chromatographic resolution, as displayed in Figure 4.

Although many tropical cyanobacteria are carotenoid-rich, *S. caboverdiana* sp. nov. LEGE 181209 differs for its chlorophyll content. Total chlorophylls reached 108 ± 6.17 µg mg^−1^ DE, clearly surpassing the values previously reported for other species belonging to the same genus, and also isolated from the Cabo Verde environment (27.72–66.86 µg mg^−1^ DE) [26] (Table S2). Chlorophyll-*a* (3) was the major contributor, with 85.61 ± 1.70 µg mg^−1^ DE, while its derivative (5) appeared at lower levels (22.39 ± 4.46 µg mg^−1^ DE). On the other hand, total carotenoids did not surpass 72.73 ± 5.47 µg mg^−1^ DE, being among the lowest reported for tropical species of the same geographic location [26]. Of them, β-carotene (10) dominated with 42.08 ± 2.64 µg mg^−1^ DE. This is in line with the available reports on cyanobacteria carotenoids composition, where β-carotene represents the major carotene [26,32]. Regarding minor compounds, lutein (1) presented 1.80 ± 0.20 µg mg^−1^ DE, which is in the same order of values of those previously reported for species of the same genus; on the other hand, myxoxanthophyll (6) was present in lower values (3.52 ± 0.24 µg mg^−1^ DE) than those reported in the literature [26]. Zeaxanthin (4) was quantified at 8.18 ± 0.57 µg mg^−1^ DE, a value consistent with previous reports for the genus. The content of lutein and zeaxanthin reported by Morone et al. [26] from Cabo Verde isolates are in the same order of magnitude as those observed herein, even though maximum carotenoid levels reached higher values (70.47 to 186.71 µg mg^−1^ DE) in their study. β-Cryptoxanthin (7) was detected at 1.80 ± 0.42 µg mg^−1^ DE, but was absent in the previous report, which reinforces that there is species-specific pigment variability within the same geographic origin. To the best of our knowledge, this is the first report on the occurrence of this xanthophyll in the *Salileptolyngbya* genus.

Although the reports on the carotenoids profile of cyanobacteria are still limited, when comparing the results obtained herein with those reported for cyanobacteria isolated from other regions of the globe, and belonging to the same order, distinct patterns can be noticed. For instance, a survey with *Nodosilinea antarctica* LEGE 13457, a strain isolated from the Victoria Valley (McMurdo Dry Valleys, Antarctica), reported an amount of chlorophylls (417.58 µg mg^−1^ DE) almost seven times higher than that of carotenoids (63.93 µg mg^−1^ DE) [22]. On the contrary, other surveys with cyanobacteria of different genera, isolated from the extreme environment of the volcanic lake Chichonal (Mexico), reported a carotenoids profile significantly dominated by carotenoids, to the detriment of chlorophylls [33].

#### 2.2.2. Phycobiliproteins and Chlorophylls

**Figure 5:**
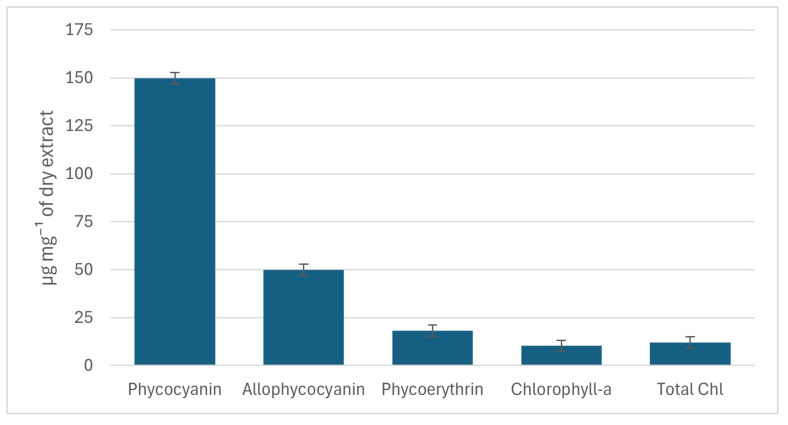
Phycobiliproteins (allophycocyanin, phycocyanin and phycoerythrin) and chlorophylls (µg mg^−1^ dry extract) quantified in the aqueous extract of *S. caboverdiana* sp. nov. LEGE 181209. TChls, total chlorophylls.

PBPs, as water-soluble molecules, were quantified spectrophotometrically only in the aqueous extract. All three main components were detected: phycocyanin (PC), phycoerythrin (PE) and allophycocyanin (APC) (Figure 5). PC, responsible for the characteristic blue coloration, was the most abundant, followed by PE, which contributes to reddish tones, whereas APC, associated with a bluish-green color, was comparatively lower. This distribution was also reflected in the coloration of the aqueous extracts as commonly reported [34].

Phycocyanin (PC) was the predominant pigment, reaching 149.83 ± 2.92 µg mg^−1^ DE, followed by APC (49.83 ± 10.15 µg mg^−1^ DE) and by PE, which was detected at lower levels (18.25 ± 7.33 µg mg^−1^ DE).

The predominance of PC, followed by PE, aligns with the canonical organization of cyanobacterial phycobilisomes, where PC is the major component of the rods and transfers excitation energy to the APC core [34]. This pigment distribution is consistent with previous reports for *Salileptolyngbya* strains and other tropical cyanobacteria [26,32,33]. The PC content obtained here is among the highest reported for other *Salileptolyngbya* strains, isolated from the same region of the globe (17.70–222.76 µg mg^−1^ DE) (Table S2) [9], supporting its potential as a high-performing PC producer. Phycoerythrin followed a similar trend, this new strain being a top producer when compared to the previously reported *Salileptolyngbya* strains isolated from the same geographic location, with values ranging from 4.03 to 14.43 µg mg^−1^ DE [9] (Table S2). This reinforces species-specific characteristics as an explanation for the different pigments profile within species of the same genera, as previously pointed out by other authors. Silva et al. [33] reported that *Tolypothrix* sp. exhibited particularly high PE levels, consistent with observations in other *Tolypothrix* species. Similarly, Soule and coworkers [35] demonstrated that *Nostoc punctiforme* predominantly accumulates PC, followed by PE and APC, a profile comparable to the one observed in the present work. Although APC levels can surpass those of PE in some cyanobacteria, this was not the case in our study, where PE consistently exceeded APC. The relatively high standard deviations observed for PE may be explained by its greater instability compared with PC and APC, as PE is prone to degradation during extraction and storage [36,37]. From a biotechnological perspective, high PC levels in *S. caboverdiana* sp. nov. LEGE 181209 support its potential to provide natural blue pigments for the food, pharmaceutical and cosmetic industries [38].

In addition to PBPs, chlorophyll-*a* and total chlorophylls (TChls) were quantified in the aqueous extract (Figure 5). *S. caboverdiana* sp. nov. LEGE 181209 exhibited 10.29 ± 2.66 µg mg^−1^ DE of chlorophyll-*a* and 12.03 ± 4.16 µg mg^−1^ DE of total chlorophylls. Chlorophyll levels were lower than that of PBPs, reflecting functional complementarity: PBPs expand light absorption into spectral regions that are less efficiently captured by chlorophylls [39]. Altogether, the aqueous extract of *S. caboverdiana* sp. nov. LEGE 181209 revealed a pigment composition dominated by PC and complemented by moderate amounts of PE, APC, and chlorophylls, reinforcing its potential for biotechnological valorization as a source of multifunctional natural pigments.

#### 2.2.3. Total Phenols and Sugars Content

The total phenolic content of the aqueous extract of *S. caboverdiana* sp. nov. LEGE 181209 was 8.77 ± 1.20 µg of gallic acid equivalents (GAE) mg^−1^ DE. Compared with the literature, the TPC value obtained herein was lower than that reported for other Cabo Verde strains (≈12–72 µg GAE mg^−1^) [26], with *Salileptolyngbya* often among the top producers. The variation is consistent with phylogeny, cultivation regime, and extraction solvent; environmental stressors such as elevated temperature can enhance phenolic accumulation [40], and organic solvents (acetone/methanol) often recover more phenolics than water [41]. Biotechnologically, phenolic-rich extracts are attractive as sustainable sources of natural antioxidants for nutraceutical, cosmetic and pharmaceutical applications. In addition to their free-radical scavenging capacity, phenolic compounds can act as preservatives, stabilizers and bioactive ingredients in functional formulations [42].

Total sugars were also quantified in the aqueous extract by the phenol–sulphuric acid method [43]. This colorimetric assay detects all carbohydrate fractions present in the extract, revealing considerable variability among strains. Total sugars concentration reached 19.71 ± 1.98 µg of glucose equivalents (GE) per mg of DE. When compared with the literature values on the same basis, our total sugars content was lower than those previously reported for other Cabo Verde strains (≈77–351 µg mg^−1^ GE per mg of DE) [9]. Among them, *Salileptolyngbya* sp. LEGE 181187 (295.75 µg mg^−1^ per mg of DE) stood out as one of the top producers. Interestingly, these values are comparable to those reported in the literature for *Nostoc muscorum* (≈320 µg mg^−1^ GE per mg of DE), a species recognized as a high carbohydrate producer.

In cyanobacteria, carbohydrates fulfill dual roles: they act as intracellular energy reserves, mainly in the form of glycogen and sucrose, and are also secreted as extracellular polysaccharides (EPS), which enhance tolerance to environmental stress and mediate ecological interactions such as adhesion and biofilm development [44]. This metabolic flexibility reflects the strong influence of strain-specific traits and cultivation conditions on sugar accumulation patterns [45]. These isolates remain valuable given EPS applications (stabilizers, thickeners, bioactives) and potential as feedstocks for biofuels/bioplastics [46]. In this sense, although *S. caboverdiana* sp. nov. LEGE 181209 presents lower sugars content than other species of the same genera, when combined with its high pigments production, this reinforces its biotechnological value for integrated valorization strategies [47,48].

### 2.3. Cytotoxicity of Cyanobacteria Extracts

The evaluation of cytotoxicity is a crucial step in characterizing natural extracts, as it ensures the absence of toxic compounds that could compromise applications in health-related fields. Cytotoxicity assays provide insights into the safety of bioactive extracts, reduce the need for animal testing, support sustainable use, and validate their potential for pharmaceutical, cosmetic, or nutraceutical purposes. Human keratinocytes (HaCaT) are particularly relevant for predicting compatibility with the skin barrier, making them appropriate models for dermatological applications [9]. In this study, the cytotoxicity of *S. caboverdiana* sp. nov. LEGE 181209 extracts was assessed in HaCaT cells after 24 h and 48 h of exposure (Figure S2). For the acetone extract, a significant reduction in cells viability was detected at the highest concentration tested (200 µg mL^−1^), but only for an incubation period of 48h (*p* > 0.05) (Figure S2a). For lower concentrations (12.5–50 µg mL^−1^), an increase in cells viability was observed (*p* < 0.05), which could be correlated with the stimulation of cell proliferation by the extracts. However, accounting for the high standard deviations inherent to the assay, and considering that there is a tendency for the reduction of cells viability with increasing extracts concentration, this assumption would need further analysis to be confirmed. A comparable profile was observed for the aqueous extract, where most concentrations did not induce significant decreases in cell viability (*p* > 0.05) (Figure S2b). In general, HaCaT cells exposed to *S. caboverdiana* sp. nov. LEGE 181209 extracts maintained viability comparable to the control across the tested concentration range, showing only occasional deviations without a clear dose–response trend (*p* < 0.05). Overall, both acetonic and aqueous extracts exhibited low or absent cytotoxicity in HaCaT cells, consistent with previous studies with strains isolated from Cabo Verde, where extracts rarely induced marked cytotoxic effects in mammalian cell lines [9,26]. Notably, the present results are in line with those reported for strains of the same genera in earlier studies. *Salileptolyngbya* strains described by Morone et al. [9] maintained cell viabilities above 80–90% with no significant cytotoxicity, a profile similarly observed for *S. caboverdiana* LEGE 181209, LEGE 181216, and LEGE 181219. Altogether, these findings reinforce the low cytotoxic potential of these genera and support the suitability of these extracts for subsequent analyses.

#### Toxin Analysis

PCR assays targeting microcystin (*mcyA*) and anatoxin (*anaC*) biosynthetic genes were negative for all isolates (Figure S3), demonstrating the absence of these cyanotoxin pathways and supporting their potential for safe biotechnological exploitation. Despite this, research into other, less common toxins, should not be ruled out.

## 3. Materials and Methods

### 3.1. Isolation and Morphological Analysis

**Figure 6:**
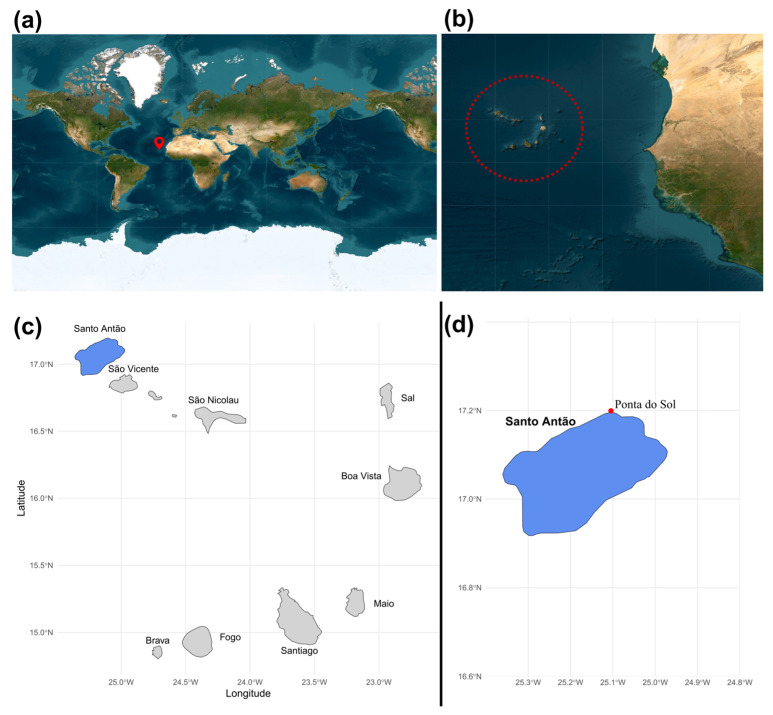
(**a**) Cabo Verde Archipelago location. (**b**) The dotted circle marks the islands composing the Cabo Verde archipelago. (**c**) Map of Cabo Verde Islands where the sample was collected. (**d**) Location of the sampling site at Santo Antão.

The cyanobacteria strain LEGE 181209 was isolated from a cyanobacterial mat collected from a rock surface on a pier wall in the coastal marine ecosystem of Cabo Verde (Figure 6a,b), specifically on the island of Santo Antão (17° 12.28′ N, 25° 05.53′ W) (Figure 6c,d) [49]. For that, filaments from the natural sample were inoculated into 24-well plates with Z8 medium (containing MgSO_4_·7H_2_O, NaNO_3_, Ca(NO_3_)_2_·4H_2_O, NH_4_Cl, Na_2_CO_3_, FeEDTA solution and micronutrients; Sigma-Aldrich, St. Louis, MO, USA), enriched with 10 μg L^−1^ of vitamin B12, and supplemented with 25 g L^−1^ of synthetic sea salts (Tropic marine, Berlin, Germany) (Z825) [50]. After isolation, the resultant biomass was transferred to a 40 mL flask with fresh Z825 medium and maintained in the Blue Biotechnology and Ecotoxicology Culture Collection (LEGE-CC) at 22 °C under a 12:12 h light: dark cycle and at a light intensity of 25 μmol photons m^−2^ s^−1^. From this culture, microphotographs and measurements were taken using a Leica DMLB microscope equipped with a camera and LEICA LAS version 4.12.0 image analysis software.

### 3.2. Scanning Electron Microscopy (SEM)

SEM analysis was used to examine cell surface morphology [51], following the protocol described by Morais et al. [29]. Cells were harvested by centrifugation and fixed overnight (12 h) at 4 °C in a final solution of 2% glutaraldehyde (for electron microscopy, VWR BDH Chemicals, Prague, Czech Republic) prepared in 50mM sodium cacodylate buffer, pH 7.4 (Electron Microscopy Sciences, PA, USA). After being postfixed overnight, cells were washed once in a double-strength sodium cacodylate buffer. Dehydration was performed through a graded ethanol series in deionized water (*v*/*v*) of 25%, 50%, 75%, and 100%, with each step lasting 5 min (Ethanol Absolute, Molecular Biology Grade, Fisher BioReagents, Loughborough, United Kingdom). Between steps, cells were collected by centrifugation at 11,000× *g* for 1 min at room temperature (Micro Star 17R, VWR, Radnor, PA, USA). In the final step, 500 μL of 100% ethanol was added to fully submerge the sample. Critical-point drying was used (CPD 7501, Polaron Range, Warsaw, Poland) to preserve the sample’s ultrastructure. Samples were coated with a gold/palladium (Au/Pd) thin film in adhesive carbon tape by sputtering, using the SPI Module Sputter Coater equipment for 80 s and a 15 mA current. SEM/EDS analysis was performed using a high-resolution (Schottky) Environmental Scanning Electron Microscope with X-Ray Microanalysis and Electron Backscattered Diffraction analysis: FEI Quanta 400 FEG ESEM/EDAX Genesis X4M. Images were taken with the SEM software version 4.1.10.2127 at an acceleration voltage of 15kV with the Everhart–Thornley detector (ETD) for secondary electrons (SE) and Backscattered Electron Detector (BSED) for backscattered electrons (BSE).

### 3.3. Phylogenetic Analysis

#### 3.3.1. DNA Extraction, PCR Amplification and Sequencing

The cyanobacterial cells were harvested from the culture, and the total genomic DNA (gDNA) of the strains were extracted using the PureLink Genomic DNA kit (Invitrogen, Waltham, MA, USA), following the manufacturer’s instructions provided for Gram-negative bacteria. The 16S–23S rRNA region was amplified via PCR using primers 27SF [52] and 23SR [53], following previously described amplification conditions [54]. Additionally, independent PCR reactions were performed to assess the presence of toxin-synthesis-related genes encoding microcystin (*mcyA*) and anatoxin (*anaC*), using specific primer sets and PCR programs as previously described [55]. To assess the presence and quality of the DNA obtained from extraction and PCR, electrophoresis on 1% (*w*/*v*) and 1.5% (*w*/*v*) agarose gels, stained with SYBR Safe DNA gel stain (Invitrogen by Thermo Fisher Scientific, Sunnyvale, CA, USA) were performed. The confirmation of DNA was based on the presence of clear bands observed in the gel. The obtained sequence was deposited in GenBank (National Center for Biotechnology Information, NCBI) under the ID.PQ454227.

#### 3.3.2. Phylogenetic Analysis

To determine the phylogenetic position of *S. caboverdiana* sp. nov. LEGE 181209 and its identification at the species level, the 16S rRNA gene sequence of this strain was aligned with sequences of cyanobacterial reference strains from the orders Leptolynbyales and Nodosilineales, with additional sequences retrieved from GenBank (NCBI) by BLAST (version 2.12.0). This alignment contained 90 sequences. Then, the phylogenetic trees were built using Maximum Likelihood (ML) and Bayesian Inference (BI) methods. GTR+G+I evolutionary model was selected by MEGA11: Molecular Evolutionary Genetics Analysis version 11 [56], according to the AIC criterium. The robustness of ML tree was estimated by bootstrap percentages, using 1000 replications using IQ-Tree online version v1.6.12 [57]. The BI tree was constructed in two independent runs, with four chains each, for 5 × 10^6^ generations, with burnin fraction set to 0.25, sample frequency 1000, using MrBayes [58] in Cipres Gateway [59]. The processing and visualization of these trees were made using FigTree v1.4.4 [60].

For all analyses, the sequences were aligned using MAFFT [61] and the outgroup used was Gloeobacter violaceus PCC 8105 (AF132791). The alignment was performed with the full length of the 16S rRNA gene sequences of the strains in the trees. The 16S rRNA gene and 16S–23S ITS p-distances were calculated using MEGA11. The 16S–23S ITS secondary structures of D1-D1’, Box B and V3 helices were folded using MFold [62], according to Lukesova et al. [63].

### 3.4. Cultures Scale-Up and Collection of Biomass

The cyanobacterial strain *S. caboverdiana* sp. nov. LEGE 181209 was isolated from the biological samples. The biomass was manipulated under aseptic conditions in a laminar-flow cabinet. The culture was scaled up until it reached 4 L total volume. The 4 L culture was maintained in photobioreactors equipped with aeration systems providing continuous air flow, including both inlet and outlet streams, to ensure optimal conditions for cyanobacterial growth. The strain was cultivated in Z825 medium [50] and the culture was maintained in the Bioterium of Aquatic Organisms (BOGA) of CIIMAR at a constant temperature (25 °C ± 2 °C) under continuous aeration, with a light intensity of 10–30 μmol photons m^−2^ s^−1^ and a photoperiod of 14 h light/10 h dark. Fresh biomass was harvested by filtration using nylon mesh filters (Kunststoff-Analysensieb, DIN 4197) with 11 µm pore sizes, selected according to the strain morphology, and an extra step was added, consisting of three washes with deionized water to remove the salts. The filtered biomass was immediately frozen, freeze-dried (Telstar LyoQuest), and stored at −20 °C until analysis.

### 3.5. Preparation of Extracts

Sequential acetone and aqueous extracts were prepared from the dry cyanobacteria biomass. In order to increase surface area and improve solvent penetration, the biomass was finely pulverized prior to extraction.

#### 3.5.1. Acetone Extract

Acetone extraction was performed using 0.5 g of dry biomass, which was immersed in 40 mL acetone and subjected to ultrasound-assisted extraction with an MS-72 probe (Bandelin Sonoplus, Berlin, Germany) for 5 min. The sample was kept on ice throughout the procedure to prevent temperature increases caused by sonication, thereby preserving thermolabile compounds and minimizing potential oxidative degradation. Subsequently, centrifugation was employed to remove cellular debris at 5000× *g* for 5 min at 4 °C using a Thermo Scientific™ HERAUS Megafuge™ 16R centrifuge (Waltham, MA, USA). The supernatant was filtered through 0.22 µm sterile syringe filters (FilterBio, Nantong, China). The biomass was re-extracted four times; filtrates were combined and evaporated under reduced pressure using a BUCHI R-210 Rotary Evaporator (Cambridge, MA, USA) set at 200 mbar, with the water bath at 30 °C and the condenser at −8 °C, to gently remove the solvent without degrading the extracted compounds. The residue was weighed for yield (8.4%), and stored at −20 °C until chemical and biological analyses.

#### 3.5.2. Water Extract

Following acetone extraction, the residual biomass was left in a fume hood overnight to evaporate any remaining solvent. The dry pellet was then extracted with 40 mL of ultrapure water following the same procedure; this was repeated four additional times. After extraction, the mixture was centrifuged, and the supernatants collected, frozen and lyophilized. Dried extracts were weighed for yield (60%) and stored until analysis.

### 3.6. Chemical Analysis

#### 3.6.1. Carotenoids Profiling by HPLC-PDA

Dried acetone extract was resuspended in HPLC-grade acetone to 5 mg mL^−1^. Pigments profile was determined using a Waters Alliance 2695 high-performance liquid chromatography (HPLC) system equipped with a photo diode-array (PDA) detector (USA), following a previously described method reported by Morone et al. [64]. Separation used a YMC Carotenoid C30 (250 × 4.6 mm, 5 μm) column, at 25 °C using a column heater (Waters Corporation, Milford, CT, USA). The mobile phase consisted of methanol (A) and methyl tert-butyl ether (B) (VWR Prolabo), applying the following gradient elution: starting with 5% B, followed by 10% B at 5 min, 18% B at 20 min, 30% B at 28 min, 50% B from 31 to 37 min, 80% B from 38 to 47 min, and returning to 5% B from 48 to 50 min. The flow rate was 0.9 mL min^−1^, with an injection volume of 5 μL. Chromatographic data were processed with Empower^TM^2^®^ software (version 2) (Waters Corporation, Milford, MA, USA). Spectra were collected between 250 and 750 nm. Compounds were identified by retention times and UV–Vis spectra with those of authentic standards. Carotenoids were quantified by peak absorbance at 450 nm and compared with calibration curves of the respective external standards. The following standards were used: lutein, β-carotene, chlorophyll-*a*, zeaxanthin and β-cryptoxanthin (Extrasynthese, Genay, France; Sigma-Aldrich, St. Louis, MO, USA; DHI, Hørsholm, Denmark). Unidentified carotenoids were quantified as β-carotene, the most representative carotene; β-carotene derivatives as β-carotene; chlorophyll derivative as chlorophyll-*a*, the major cyanobacteria chlorophyll; and myxoxanthophyll as zeaxanthin, the most representative xanthophyll in this study. Calibration curves were performed with five different concentrations of standards, selected as representative of the range of compounds concentration expected in the samples. Calibration plots, *r*^2^, LOD and LOQ values are shown in Table 2.

#### 3.6.2. Phycobiliproteins and Chlorophylls Profiling by Spectrophotometry

Polar pigments in aqueous extract were quantified spectrophotometrically. The extract was resuspended in ultrapure water (for PBPs) or acetone (for chlorophylls), to a final concentration of 0.5 mg mL^−1^, and analyzed in duplicate. Results were expressed in µg of pigment per mg of DE [65]. Absorbance was read at 562, 615, and 652 nm, and concentrations were calculated using standard equations as previously described [66]:

(1) $$Phycocyanin\left(PC\right)=\frac{\left(A_{615nm}-0.474\times A_{652nm}\right)}{5.34}$$

(2) $$Allophycocyanin\left(APC\right)=\frac{\left(A_{652nm}-0.208\times A_{615nm}\right)}{5.09}$$

(3) $$Phycoerythrin\left(PE\right)=\frac{\left(A_{562nm}-2.41\times PC-0.849\times APC\right)}{9.62}$$

For chlorophylls, absorbance was read at 630, 647, 664, and 691 nm. Concentrations were calculated according to the following equations [66]:

(4) $$Chla=-0.3319\left(A_{630nm}\right)-1.7485\left(A_{647nm}\right)+11.9442\left(A_{664nm}\right)-1.4306\left(A_{691nm}\right)$$

(5) $$TotalChl=21.3877\left(A_{630nm}\right)+10.3739\left(A_{647nm}\right)+5.3805\left(A_{664nm}\right)+5.5309\left(A_{691nm}\right)$$

#### 3.6.3. Total Phenolic Content (TPC) by Folin–Ciocalteu Method

The total phenolic content was determined by the colorimetric Folin–Ciocalteu method, as previously described [67], with slight modifications.

The aqueous extract was resuspended in 80% ethanol to a final concentration of 10 mg mL^−1^; then, 25 µL of extract was mixed with 500 µL deionized water, 100 µL sodium carbonate solution (Na_2_CO_3_, 75 g L^−1^), and 25 µL Folin–Ciocalteu reagent (Sigma-Aldrich). Blanks contained 25 µL of 80% ethanol instead of reagent. Mixtures were incubated 60 min at room temperature in the dark. Then, 200 µL from each reaction mixture was transferred to a 96-well plate and its absorbance was read at 725 nm. Gallic acid was used to build a calibration curve (*y* = 2.7771*x* − 0.0164, *r*^2^ = 0.9998). Results were expressed in µg of GAE per mg of DE, as mean ± standard deviation of two independent experiments.

#### 3.6.4. Determination of Total Sugars by the Phenol–Sulfuric Method

Total sugars were quantified by the phenol–sulfuric acid method [43], with slight modifications. The method detects nearly all classes of carbohydrates, once sulfuric acid added to the sample hydrolyzes complex carbohydrates into their constituent monosaccharides. The aqueous extract was resuspended in 80% ethanol. Standard solutions of glucose monohydrate, ranging from 50 to 1000 mgL^−1^, were prepared to build the glucose calibration curve (*y* = 1.4616*x* + 0.0252, *r*^2^ = 0.9985). 100 µL of standard, sample, or sample vehicle (blank) were mixed with 500 µL of H_2_SO_4_ and 100 µL of 20% phenol solution. Then, 200 µL from each mixture was transferred to a 96-well plate and incubated in the dark for 30 min, after which the absorbance was read at 490 nm. Analyses were performed in triplicate and expressed as mg GE per mg of DE.

### 3.7. Cytotoxicity to Human Keratinocytes (HaCaT)

To evaluate the safety profile of the acetone and aqueous cyanobacterial extracts, an in vitro cytotoxicity screening was conducted with human keratinocytes (HaCaT, ATCC).

#### 3.7.1. Cells Culturing

Cells were thawed and maintained for one week prior to assays to allow recovery. During this period, only medium replacement was performed. Cells were cultured in high-glucose Dulbecco’s Modified Eagle Medium (DMEM, Gibco) supplemented with 10% fetal bovine serum (FBS, Gibco), 1% penicillin–streptomycin (100 IU mL^−1^ and 10 mg mL^−1^, respectively; Gibco), and 0.1% amphotericin B (Gibco). Cultures were kept at 37 °C in a humidified atmosphere with 5% CO_2_, and the culture medium was renewed every two days. At 80–90% confluence, cells were detached with TrypLE Express Enzyme (Gibco), centrifuged (Eppendorf 5430), resuspended in DMEM, and seeded or transferred to new flasks. All procedures were performed under sterile conditions in a Thermo Scientific™ Safe 2020 laminar flow chamber (Thermo Fisher Scientific, Waltham, MA, USA).

#### 3.7.2. Cytotoxicity Assessment by the MTT Assay

Cells viability was assessed by the reduction of 3-(4,5-dimethylthiazol-2-yl)-2,5-diphenyltetrazolium bromide (MTT, 98%; Thermo Scientific), as previously described in the literature [41,68]. Cells were seeded in 96-well plates at a density of 2.5 × 10^4^ cells mL^−1^. After the 24 h adhesion period, the medium was removed and the cells were exposed for 24 and 48 h to five serial concentrations of each extract (12.5 to 200 µg mL^−1^). The acetone extract was resuspended in dimethyl sulfoxide (DMSO, Gibco) and diluted in DMEM to ensure a final DMSO concentration below 1%. The aqueous extract was resuspended in PBS and diluted in DMEM. All extracts serial dilutions were prepared from a 20 mg mL^−1^ stock. Solvent controls included 1% DMSO (acetone-extract control) and DMEM (aqueous-extract control). A 20% DMSO solution in DMEM was used as a positive control for cell death.

After exposure, 20 µL of MTT solution (1mg mL^−1^ in PBS) was added per well and plates further incubated during 3 h. The medium was removed, 100 µL DMSO was added to solubilize the purple-colored formazan crystals, and absorbance was read at 550 nm on a Synergy HT multi-detection microplate reader (BioTek, Bad Friedrichshall, Germany) with Gen5™ (version 2.0) software. Assays were conducted in quadruplicate and the results were expressed as mean ± standard deviation. Cytotoxicity was expressed as percentage (%) of cells viability relative to solvent control.

### 3.8. Statistical Analysis

For analysis, various R packages (version 4.4.0) were employed to ensure robust and accurate data interpretation. Data were first checked for normality using the Shapiro–Wilk test, implemented through the ‘shapiro.test()’ function from the ‘stats’ package. Homogeneity of variances was assessed using Levene’s test, which was conducted using the ‘levene Test()’ function from the ‘car’ package. Since the data did not follow a normal distribution, group comparisons were performed using a non-parametric bootstrap approach with 1000 iterations. The empirical p-value was calculated as the proportion of resampled mean differences greater than or equal to the observed difference. The map was created using the geom_sf() function from the ggplot2 package in R. First, the geographic data were loaded and prepared using the sf package. The ggplot() function initiated the plot, and geom_sf() was used to add the spatial data layer to the map [69].

## 4. Conclusions

Collectively, our results demonstrate that marine cyanobacterium from Cabo Verde provides a robust basis for biodiversity discovery and pigment centered biotechnology. *S. caboverdiana* sp. nov. LEGE 181209 displayed distinct ultrastructural traits, most notably conspicuous pseudovacuoles under SEM, and a divergent 16S–23S ITS region, supporting its separation at the species level. PCR screening showed no amplification of *mcyA* or *anaC*, reinforcing a safety-forward profile for downstream applications.

Sequential extraction with solvents of different polarities yielded two complementary metabolite fractions. The acetone extract was enriched in canonical carotenoids, particularly β-carotene and zeaxanthin, along with chlorophyll-*a*, whereas the aqueous extract contained abundant PBPs, predominantly PC. The balanced pigment profile, together with low cytotoxicity toward HaCaT keratinocytes, highlights the strain’s potential as a source of biocompatible natural pigments. In this regard, further studies aiming to optimize growth conditions will be essential to improve the strains’ performance regarding the production of bioactive metabolites for industrial applications.

These findings position *S. caboverdiana* sp. nov. LEGE 181209 as a distinct taxon and underscore Cabo Verde’s importance as a reservoir of novel, safe and pigment-rich cyanobacteria with promising applications in sustainable blue biotechnology.

## Figures and Tables

**Table 1:** Morphological and habitat comparisons of *Salileptolyngbya* species.

	*S. diazotrophica* [27]	*S. insularis* [28]	*S.caboverdiana* sp. nov. LEGE 181209
Trichomes	Not attenuated at the ends. Markedly constricted	Not attenuated at the ends. Constricted.	Not attenuated. Constricted
Mucilaginous envelope	Firm, thin sheaths	Absent	Absent
Cell shape	Longer than width	2.8× longer than width. Translucent septa between cells.	Isodiametric longer or shorter than width.
Cell measurements	1.5–2.4 µm long × 0.9–1.5 µm wide.	1.7–3.1 μm long × 0.6–1.0 μm wide	1.3–1.9 μm long × 1.35–1.60 μm wide
Cell content	Homogeneous. Gasvesicles absent.	Homogeneous. Gasvesicles absent.	Pseudovacuoles visible only under SEM. Gas vesicles absent
Habitat	Marine, planktonic	Marine, benthic, epipsamic.	Marine, growing on rocks of a Pier wall.

**Table 2:** Calibration curves of authentic standards used for quantification of different carotenoids and chlorophylls.

Standards	Calibration Curve	*r* ^2^	LOD ^a^	LOQ ^b^
Lutein	*y* = 52,314,156.52*x* – 197,720.04	0.9900	5.6570	17.1425
β-Carotene	*y* = 34,727,408.00*x* – 96,390.00	1.0000	3.6904	11.1830
Chlorophyll-*a*	*y* = 5,242,773.3333*x* + 26,570.00	0.9981	8.9087	26.9960
Zeaxanthin	*y* = 66,258,082.7826*x* + 130,225.43	0.9997	1.2530	3.7970
β-Cryptoxanthin	*y* = 17,708,546.7826*x* + 68,409.87	0.9977	6.4075	19.4167

^a^ limit of detection (µg mL^−1^); ^b^ limit of quantification (µg mL^−1^).

## Data Availability

Data will be made available upon request to the corresponding author.

## Supplementary Materials

The following supporting information can be downloaded at https://www.mdpi.com/article/10.3390/md24010029/s1: Figure S1. Backscattered electron (BSED) SEM analysis showing the elemental composition profile of a pseudovacuole. The BSED mode provides contrast based on atomic number, revealing variations in elemental distribution within the intracellular compartment. Figure S2. Viability of keratinocytes (HaCaT) after 24 h and 48 h of incubation with cyanobacteria acetonic (a) and aqueous (b) extracts, at concentrations ranging from 12.5 to 200 µg mL^−1^. Results are expressed as % of MTT reduction relative to the untreated control. DMSO (20%) was used as the positive control for cytotoxicity. Results are presented as mean ± SD of at least three independent experiments, performed in quadruplicate. Statistical differences are indicated at * *p* < 0.05 (non-parametric bootstrap resampling, 1000 iterations). Figure S3. PCR gels showing absence of *mcyA* (a) and *anaC* (b) amplification. Positive control (+), *Leptothoe* sp. LEGE 181201; *Salileptolyngbya caboverdiana* sp. nov. LEGE 181209; Geminocystaceae cyanobacterium LEGE 181210; *Salileptolyngbya* sp. LEGE 181216; *Salileptolyngbya* sp. LEGE 181219; Leptolyngbyaceae cyanobacterium LEGE 181156. Table S1. 16S rRNA gene similarity (p-distance) matrix comparing *Salileptolyngbya* strains [56,70]. Table S2. Comparative pigment content of the studied *Salileptolynbya* strain and related cyanobacteria [9,26,41].
