# Supplementary material for: Description of a New Marine Cyanobacterium from the Cabo Verde Archipelago: Pigments Profile and Biotechnological Potential of Salileptolyngbya caboverdiana sp. nov
Source: Mar Drugs. 2026 Jan 8;24(1):29. doi: 10.3390/md24010029 (PMC12842673; doi:10.3390/md24010029)
Supplement: Supplementary file 1 [file marinedrugs-24-00029-s001.zip › Table S2.pdf]

Table S2. Comparative pigment content of the studied *Salileptolynbya* strain and related cyanobacteria.[1]

Strains	Total carotenoids	Phycocyanin	Allophycocyanin	Phycoerythrin	Chlorophyll-*a*	Reference
***Salileptolyngbya caboverdiana* sp. nov.**	72.73 ± 5.47	149.83 ± 2.92	49.83 ± 10.15	18.25 ± 7.33	10.29 ± 2.66	
Salileptolyngbya sp. LEGE 181184	115.57 ± 0.17	220.75 ± 4.18	35.59 ± 1.84	14.43 ± 0.40	42.89 ± 0.4	[1,2]
Salileptolyngbya sp. LEGE 181201	106.45 ± 0.94	17.70 ± 0.28	6.41 ± 0.44	67.34 ± 0.18	–	[1,2]
Salileptolyngbya sp. LEGE 181187	159.35 ± 0.98	69.69 ± 0.67	9.76 ± 0.10	4.03 ± 0.32	58.25 ± 2.45	[1,2]
Salileptolyngbya sp. LEGE 181150	123.26 ± 0.85	222.76 ± 0.79	57.29 ± 0.17	17.31 ± 0.11	14.77 ± 1.21	[2,3]
Salileptolyngbya sp. LEGE 181158	113.81 ± 0.81	194.43 ± 0.39	44.52 ± 0.34	17.42 ± 0.22	9.42 ± 1.01	[2,3]

[1]Data are reported as mean ± SD (where available) in μg mg^{-1}; not-determined values are denoted by "–".

References

1. Morone, J.; Hentschke, G.; Pinto, E.; Morais, J.; Cruz, P.; Vasconcelos, V.; Martins, R.; Lopes, G. Carotenoids from Cyanobacteria Modulate INOS and Inhibit the Production of Inflammatory Mediators: Promising Agents for the Treatment of Inflammatory Conditions. *Algal Res* 2024, *83*, 103729, doi:10.1016/j.algal.2024.103729.

2. Morone, J.; Hentschke, G.S.; Oliveira, I.B.; Vasconcelos, V.; Martins, R.; Lopes, G. Secondary Metabolites of Cyanobacteria from Cape Verde Archipelago Act as NO Donors with Potential Application in Dermatology and Cosmetics. *Algal Res* 2025, *86*, 103952, doi:10.1016/j.algal.2025.103952.

3. Morone, J.; Lopes, G.; Morais, J.; Neves, J.; Vasconcelos, V.; Martins, R. Cosmetic Application of Cyanobacteria Extracts with a Sustainable Vision to Skincare: Role in the Antioxidant and Antiaging Process. *Mar Drugs* 2022, *20*, 761, doi:10.3390/md20120761.
