# Supplementary material for: Description of a New Marine Cyanobacterium from the Cabo Verde Archipelago: Pigments Profile and Biotechnological Potential of Salileptolyngbya caboverdiana sp. nov
Source: Mar Drugs. 2026 Jan 8;24(1):29. doi: 10.3390/md24010029 (PMC12842673; doi:10.3390/md24010029)
Supplement: Supplementary file 1 [file marinedrugs-24-00029-s001.zip › Figure S1.pdf]

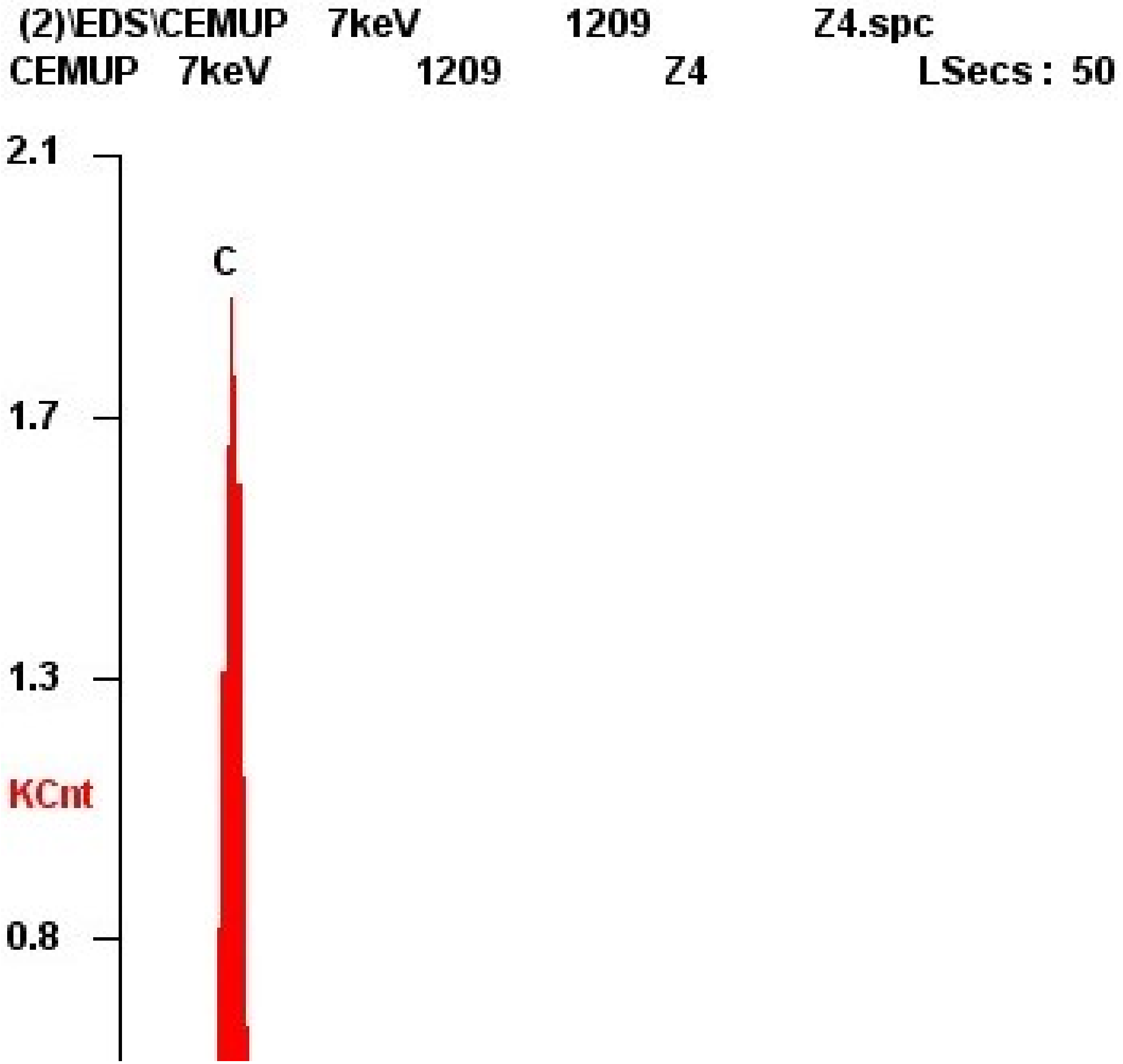

Figure S1. Backscattered electron (BSED) SEM analysis showing the elemental composition profile of a pseudovacuole. The BSED mode provides contrast based on atomic number, revealing variations in elemental distribution within the intracellular compartment.
