# Supplementary material for: Description of a New Marine Cyanobacterium from the Cabo Verde Archipelago: Pigments Profile and Biotechnological Potential of Salileptolyngbya caboverdiana sp. nov
Source: Mar Drugs. 2026 Jan 8;24(1):29. doi: 10.3390/md24010029 (PMC12842673; doi:10.3390/md24010029)
Supplement: Supplementary file 1 [file marinedrugs-24-00029-s001.zip › Figure S2.pdf]

(a) (b)

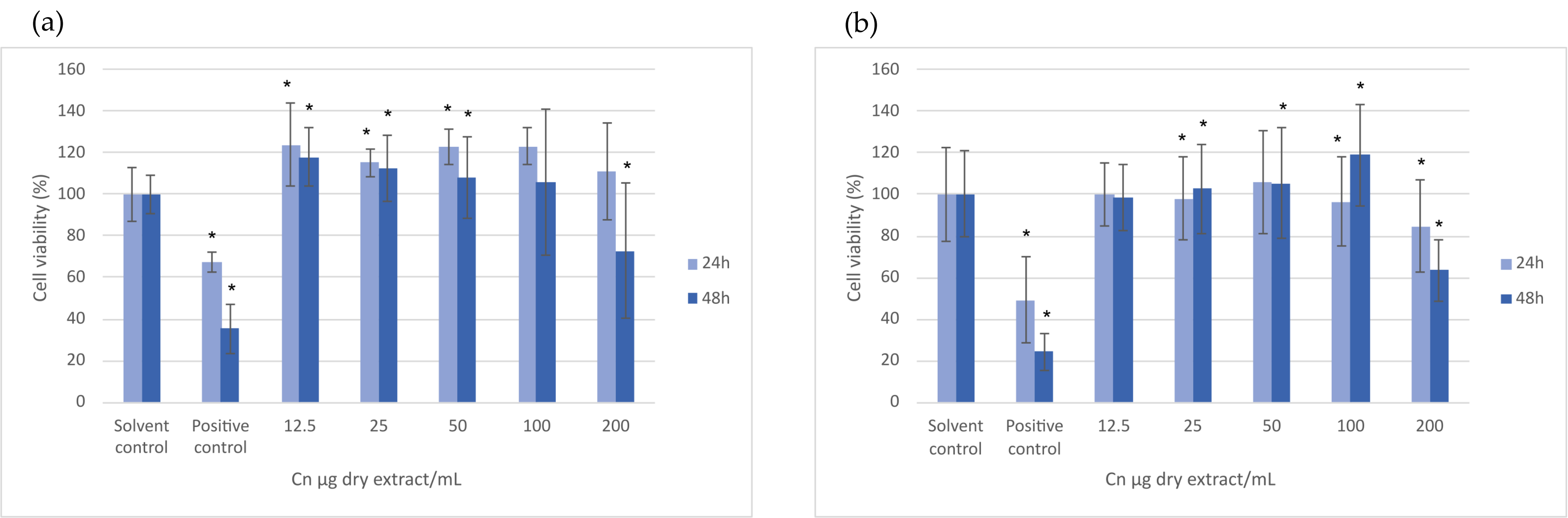

Figure S2. Viability of keratinocytes (HaCAT) after 24 h and 48 h of incubation with cyanobacteria acetonic (**a**) and aqueous (**b**) extracts, at concentrations ranging from 12.5-200 µg mL^{-1}. Results are expressed as % of MTT reduction relative to the untreated control. DMSO (20%) was used as the positive control for cytotoxicity. Results are presented as mean ± SD of at least three independent experiments, performed in quadruplicate. Statistical differences are indicated at *p<0.05 (non-parametric bootstrap resampling, 1000 iterations).
