# Supplementary material for: Description of a New Marine Cyanobacterium from the Cabo Verde Archipelago: Pigments Profile and Biotechnological Potential of Salileptolyngbya caboverdiana sp. nov
Source: Mar Drugs. 2026 Jan 8;24(1):29. doi: 10.3390/md24010029 (PMC12842673; doi:10.3390/md24010029)
Supplement: Supplementary file 1 [file marinedrugs-24-00029-s001.zip › Figure S3.pdf]

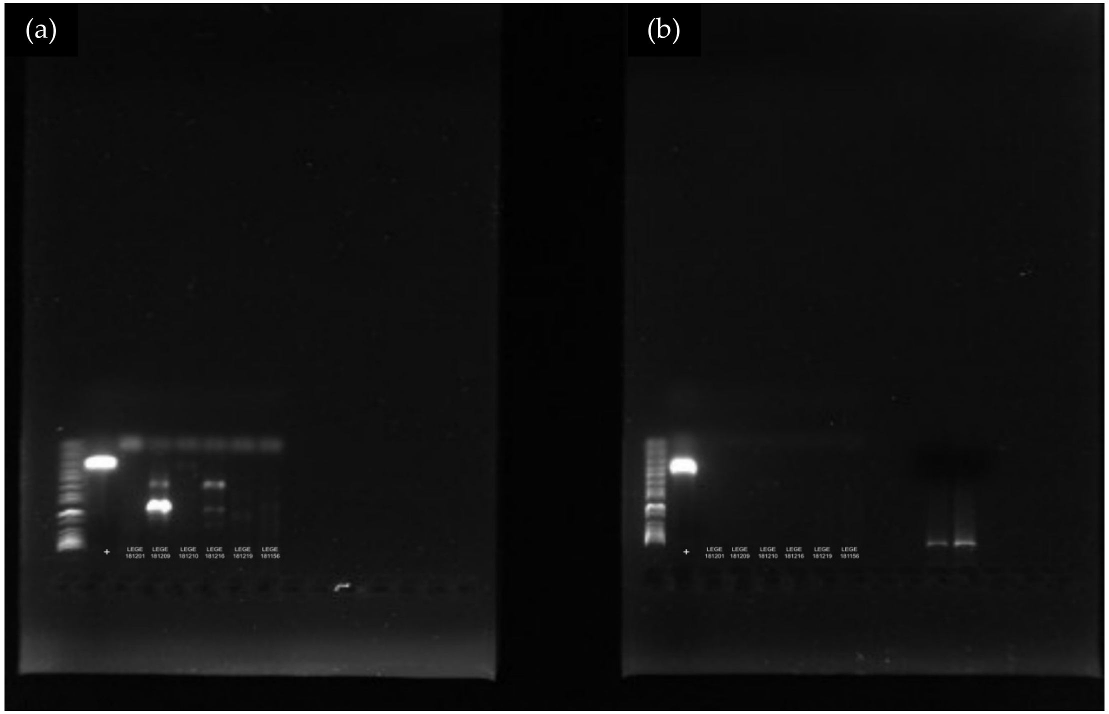

Figure S3. PCR gels showing absence of mcyA (**a**) and anaC (**b**) amplification. Positive control (+); *Leptothoe* sp. LEGE 181201; *Salileptolyngbya caboverdiana* sp. nov. LEGE 181209; Geminocystaceae cyanobacterium LEGE 181210; *Salileptolyngbya* sp. LEGE 181216; *Salileptolyngbya* sp. LEGE 181219; Leptolyngbyaceae cyanobacterium LEGE 181156.
