# Supplementary material for: Description of a New Marine Cyanobacterium from the Cabo Verde Archipelago: Pigments Profile and Biotechnological Potential of Salileptolyngbya caboverdiana sp. nov
Source: Mar Drugs. 2026 Jan 8;24(1):29. doi: 10.3390/md24010029 (PMC12842673; doi:10.3390/md24010029)
Supplement: Supplementary file 1 [file marinedrugs-24-00029-s001.zip › Table S1_updated.pdf]

Table S1. 16S rRNA gene similarity (p-distance) matrix comparing *Salileptolyngbya* strains*.

1	*S. insularis*	LEGE 181150 PQ454229																					
2	*S. insularis*	LEGE 181187 PQ454231	100,00																				
3	*S. insularis*	LEGE 181149 PQ515100	100,00	100,00																			
4	*S. insularis*	LEGE 181190 PQ454230	100,00	100,00	100,00																		
5	*S. insularis*	LEGE 181212 PQ454232	100,00	100,00	100,00	100,00																	
6	*S. insularis*	LEGE 181158 PQ515097	99,68	99,68	99,68	99,68	99,68																
7	*S. insularis*	LEGE 181198 PQ515098	99,60	99,60	99,60	99,60	99,60	99,84															
8	*S. insularis*	LEGE 181205 PQ515099	99,76	99,76	99,76	99,76	99,76	99,92	99,84														
9	*S. insularis*	LEGE 181184 PQ515094	99,44	99,44	99,44	99,44	99,44	99,60	99,52	99,68													
10	*S. insularis*	*Salileptolyngbyai insularis* ALCB132760 MW024863	99,36	99,36	99,36	99,36	99,36	99,52	99,52	99,52	99,68												
11	*Salilepolyngbya* sp. (Clade A)	LEGE 181194 PQ515101	97,48	97,48	97,48	97,48	97,48	97,56	97,56	97,48	97,48	97,48											
12	*Salilepolyngbya* sp. (Clade A)	LEGE 171504 PV275272	97,97	97,97	97,97	97,97	97,97	98,30	98,30	98,22	98,22	98,14	99,28										
13	*Salilepolyngbya* sp. (Clade A)	LEGE 181219 PQ515093	98,79	98,79	98,79	98,79	98,79	99,11	98,95	99,03	99,03	98,95	98,46	99,20									
14	*Salilepolyngbya* sp.	LEGE 181216 PQ515092	94,75	94,75	94,75	94,75	94,75	94,75	94,83	94,84	95,18	94,92	94,76	94,49	94,84								
15	*Salilepolyngbya* sp. (Clade B)	Leptolyngbya sp HBC8 EU249119	96,22	96,22	96,22	96,22	96,22	96,39	96,30	96,47	96,47	96,30	96,31	96,39	96,89	94,68							
16	*Salilepolyngbya* sp. (Clade B)	Leptolyngbya WR6 MH666137	95,97	95,97	95,97	95,97	95,97	96,13	96,14	96,13	96,14	96,14	96,56	96,56	96,64	94,08	98,79						
17	*Salilepolyngbya* sp.	Leptolyngbya CCAP1442 HE975019	96,05	96,05	96,05	96,05	96,05	95,97	95,97	95,97	96,14	96,31	96,65	96,31	96,39	94,60	96,90	97,48					
18	*S. diazotrophica*	*Salileptolyngbya diazotrophicum* SCSIO43686 MF614799	95,97	95,97	95,97	95,97	95,97	96,05	95,97	96,14	96,48	96,14	96,31	95,89	96,31	95,37	96,81	97,31	97,39				
19	*Salilepolyngbya* sp. (Clade C)	LEGE 181195 PQ515095	97,72	97,72	97,72	97,72	97,72	97,89	97,80	97,97	98,13	97,80	96,72	96,98	97,48	95,02	96,13	96,05	95,88	96,30			
20	*Salilepolyngbya* sp. (Clade C)	LEGE 181217 PQ515096	97,56	97,56	97,56	97,56	97,56	97,72	97,72	97,72	97,89	97,72	96,72	96,81	97,31	95,02	96,05	96,30	96,14	96,38	99,76		
21	*Salilepolyngbya* sp. (Clade C)	LEGE 181222 PQ454228	97,56	97,56	97,56	97,56	97,56	97,72	97,72	97,72	97,89	97,72	96,72	96,81	97,31	95,02	96,05	96,30	96,14	96,38	99,76	100,00	
22	*S. caboverdiana* sp. nov.	LEGE 181209 PQ4542270	97,97	97,97	97,97	97,97	97,97	97,97	97,97	97,97	97,64	97,81	97,06	97,06	97,89	94,66	96,47	96,22	96,47	96,80	97,72	97,89	97,89

*The number of base substitutions per site from between sequences are shown. Analyses were conducted using the Maximum Composite Likelihood model [1]. This analysis involved 22 nucleotide sequences. All positions containing gaps and missing data were eliminated (complete deletion option). There were a total of 1250 positions in the final dataset. Evolutionary analyses were conducted in MEGA11 [2]

1. Tamura K., Nei M., and Kumar S. (2004). Prospects for inferring very large phylogenies by using the neighbor-joining method. Proceedings of the National Academy of Sciences (USA) 101:11030-11035.

2. Tamura K., Stecher G., and Kumar S. (2021). MEGA 11: Molecular Evolutionary Genetics Analysis Version 11. Molecular Biology and Evolution https://doi.org/10.1093/molbev/msab120.

Disclaimer: Although utmost care has been taken to ensure the correctness of the caption, the caption text is provided "as is" without any warranty of any kind. Authors advise the user to carefully check the caption prior to its use for any purpose and report any errors or problems to the authors immediately (www.megasoftware.net). In no event shall the authors and their employers be liable for any damages, including but not limited to special, consequential, or other damages. Authors specifically disclaim all other warranties expressed or implied, including but not limited to the determination of suitability of this caption text for a specific purpose, use, or application.
